# Didelphys Uterus in Pregnancy, an Uncommon Mullerian Duct Anomaly: A Case Report

**DOI:** 10.5811/cpcem.2021.7.53212

**Published:** 2021-10-19

**Authors:** Colin Jorgensen, Monika Lusiak

**Affiliations:** AMITA Health Resurrection Medical Center, Department of Emergency Medicine, Chicago, Illinois

**Keywords:** didelphys uterus, Mullerian duct anomalies

## Abstract

**Introduction:**

Didelphys uterus, or “double uterus,” is one of the rarest Müllerian duct anomalies (MDA). Due to its rarity, data are sparse on overall outcomes associated with this congenital defect, but it may be associated with several complications, both pregnancy and non-pregnancy related.

**Case Report:**

In this case, a pregnant 35-year-old female with vaginal bleeding was subsequently diagnosed with uterus didelphys by transvaginal ultrasound imaging.

**Conclusion:**

Despite its rarity, clinicians should be aware of MDAs and their associated compli-cations with pregnancy.

## INTRODUCTION

Müllerian duct anomalies (MDA) are a spectrum of congenital defects arising from the failure of fusion of the Müllerian ducts at 12–16 weeks’ embryologic development. Sources vary on incidence of these abnormalities; MDAs range between 0.5–5% of the general population.^1^ There are several classification systems of MDAs; the most widely accepted is a modified version of the initial system characterized by the American Fertility Society. Didelphys uterus (MDA class III) accounts for approximately 5% of all MDAs^2^ and arises from the complete non-fusion of both Müllerian ducts, resulting with two distinct cervices.^3^

Didelphys uterus is associated with increased rates of infertility in comparison to normal uterine anatomy, as well as dysmenorrhea or dyspareunia. Additionally, it is characterized with Herlyn-Werner-Wunderlich syndrome, a rare disorder associated with obstructed hemivagina and ipsilateral renal agenesis. Despite its rarity, the emergency physician should be aware of didelphys uterus in the patient who presents with gynecologic and obstetric complaints.

## CASE REPORT

A 35-year-old female gravida two, para one, at approximately eight weeks estimated gestational age (EGA) by last menstrual period presented to the emergency department (ED) for vaginal bleeding, abdominal cramping, and pain. The patient reported some low abdominal cramping beginning 12 hours prior to arrival, associated with bright red vaginal bleeding, which had soaked two tampons. She and her husband presented from the airport after traveling from Europe where she was stationed for military service. Prior to receiving military clearance for travel, she had completed a scheduled medical exam and transvaginal ultrasound with diagnosis of definitive intrauterine pregnancy. Her husband brought a copy of the ultrasound report. Previous delivery records were not available as the patient had given birth outside the United States, but she reported the delivery was term via caesarean section due to breech position. Her previous pregnancy and postpartum period were reportedly otherwise unremarkable. She stated that she had no other obstetric or gynecological history.

The patient’s vital signs were within normal limits on arrival and stable throughout her evaluation in the ED. Her exam was notable for midline and left-sided lower abdominal and pelvic tenderness without guarding or rigidity. Her pelvic exam was normal in external appearance and showed blood in the cervical canal with no cervical motion tenderness or cervical dilation. Her laboratory results revealed a beta human chorionic gonadotropin of 13,340 milli-international units per milliliter (mIU/mL) (reference range: 7500–225,000 mIU/mL based on patient’s corresponding EGA); rhesus positive; antibody negative; and her hemoglobin was within normal limits.

**Image 1:**
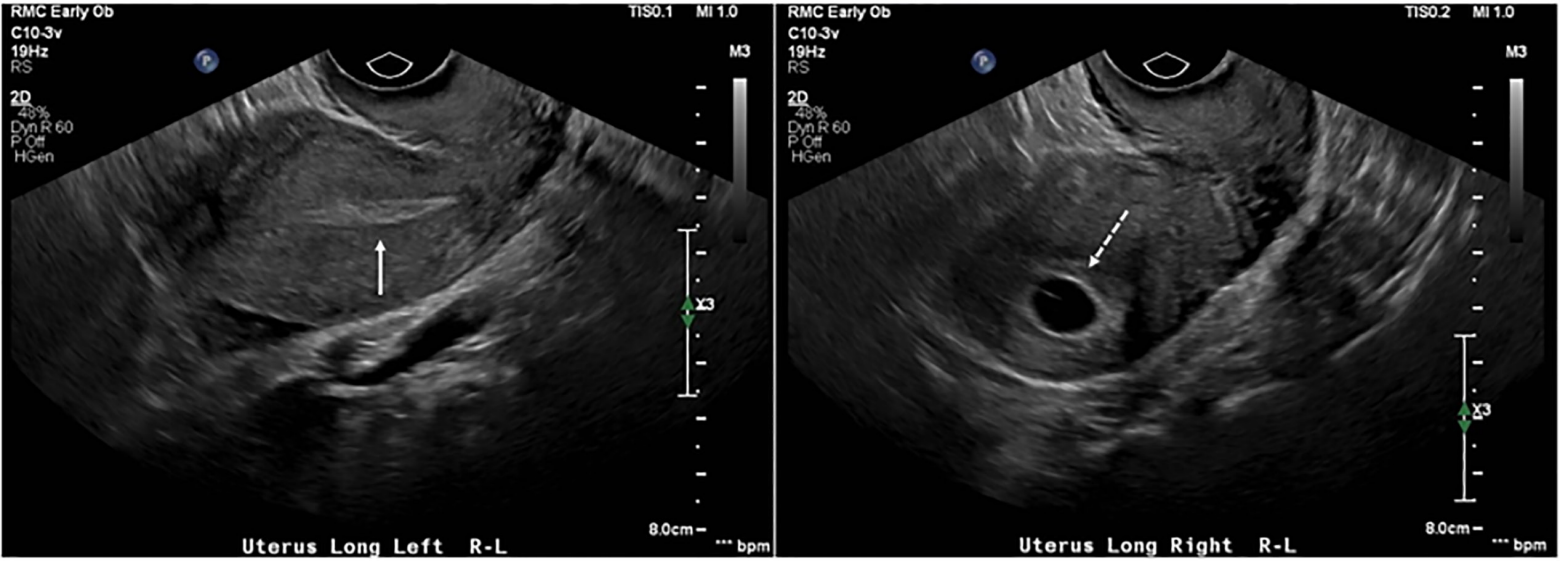
Transvaginal ultrasound. Longitudinal views of the uterus demonstrate uterus didelphys configuration with the absence of gestational sac in the left uterus (solid continuous arrow, left) and the presence of a gestational sac in the right uterus (interrupted arrow, right).

**Image 2:**
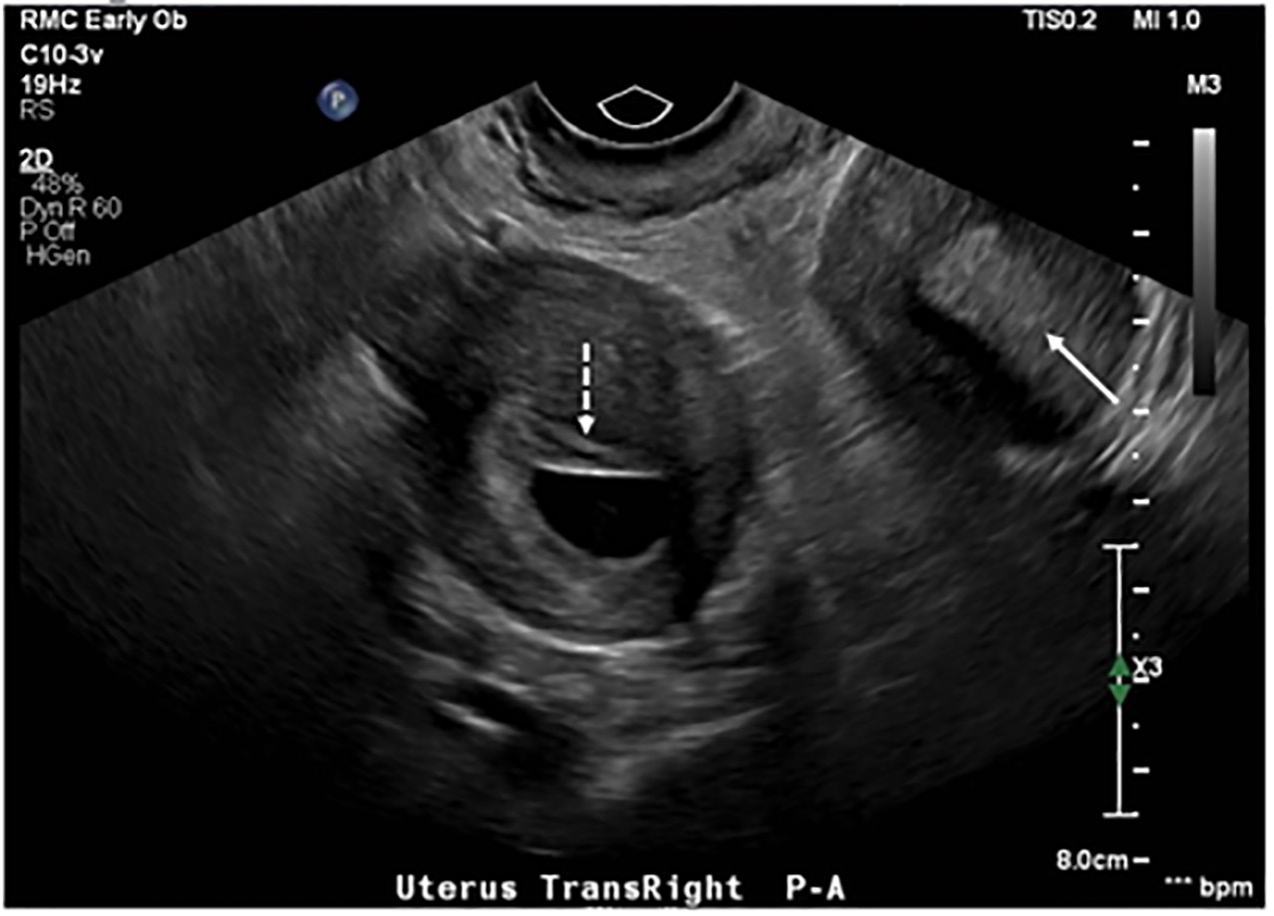
Transvaginal ultrasound. Transverse views of the uterus demonstrate uterus didelphys with both the right uterus (interrupted arrow) with gestation sac present and adjacent left uterus (solid arrow) with absence of gestation sac.

Transabdominal ultrasound was attempted, but it was difficult to obtain images consistent with visualization of a definitive intrauterine pregnancy. Subsequent transvaginal ultrasound revealed a uterine didelphys configuration with the right uterus containing a gestational sac and yolk sac that corresponded to an EGA of six weeks and two days. There was no fetal pole visualized or fetal cardiac activity. Her left uterus appeared to have a thickened endometrial stripe. (Image 1, 2)

After reviewing the findings with the patient, she did admit that she was told “something” after her first delivery but could not remember what it pertained to. The patient’s initial presentation and localization of pain to the left lower quadrant correlated with her left nongravid uterus that was likely undergoing withdrawal bleeding secondary to changes in hormone levels. During her stay in the her pain improved with two doses of morphine, and she was discharged in stable condition with close obstetrics follow-up and diagnosis of threatened abortion.

On follow-up, the patient stated that she had an otherwise uneventful pregnancy that progressed without further complications or bleeding. We postulate that alternatively to threatened abortion, bleeding may have been a result of progesterone luteal shift and withdrawal bleeding in her nongravid left uterus. In early pregnancy the corpus luteum produces progesterone to sustain a uterine environment suitable for pregnancy. The placenta takes over progesterone production within the first trimester. In the case presented here, that may have been responsible for the lack of progesterone locally within the left uterus and subsequent hormonal withdrawal bleeding as well as left-sided pain on examination.

## DISCUSSION

As noted, didelphys uterus is an uncommon MDA, accounting for approximately 5% of cases. It is often asymptomatic, but it may be discovered in association with both pregnancy- and non-pregnancy related complaints. Often diagnosed with menarche as well as with onset of sexual activity, the presence of a vaginal septation may be associated with dysmenorrhea or dyspareunia. The incidence of vaginal septation is estimated at 70%,^4^ although it was not present in this case. While exceedingly rare, Herlyn-Werner-Wunderlich syndrome is another MDA variant, which is characterized by uterine didelphys, obstructing hemivagina, and ipsilateral renal agenesis. Previous case reports have documented pediatric patients presenting to the ED with symptoms of severe abdominal pain, likely associated with dysmenorrhea with the onset of menstruation and obstructed hemivaginal septa.^5^,^6^ It is a worthwhile consideration for differential diagnosis in pediatric patients with abnormal physical examination findings such as a blind vagina and perhaps renal agenesis on initial point-of-care ultrasound for abdominal pain.

**CPC-EM Capsule**

What do we already know about this clinical entity?

*Didelphys uterus is a rare Mullerian duct anomaly (MDA) which is associated with dysmenorrhea, dyspareunia, increased infertility rates, and fetal growth restriction.*

What makes this presentation of disease reportable?

*This is a rarely documented case of first trimester vaginal bleeding in a patient with Didelphys uterus configuration.*

What is the major learning point?

*Pregnant women with MDAs should have close obstetric follow-up and be educated on increased rates of pregnancy-related complications.*

How might this improve emergency medicine practice?

*Despite their rarity, early recognition of MDAs may better define patient’s complaints when presenting to the Emergency Department.*

In cases of pregnancy, there are increased rates of infertility associated with uterus didelphys.^1^ It has also been associated with a nearly 33% miscarriage rate and 30% preterm delivery rate.^7^ Additionally, didelphys uterus is associated with fetal growth restriction.^8^ Generally, surgical management of didelphys uterus is not indicated, nor is there an indication for primary cesarean section.^9^

## CONCLUSION

Müllerian duct anomalies are seen in approximately 0.5% of the general population. Didelphys uterus is among the rarest of these anomalies. Didelphys uterus can be associated with dysmenorrhea or dyspareunia particularly in the setting of vaginal septation. Despite their rarity, it is important for the emergency physician to be mindful of MDAs, including didelphys uterus, as they are associated with increased rates of infertility and pregnancy complications.
